# Humoral Response in SARS-CoV-2 Convalescent Compared to Vaccinated Kidney Transplant Patients

**DOI:** 10.3389/ti.2021.10060

**Published:** 2022-01-18

**Authors:** Judith Schimpf, Hannelore Sprenger-Mähr, Tamara Davidovic, Karl Lhotta, Emanuel Zitt

**Affiliations:** ^1^ Department of Internal Medicine 3 (Nephrology and Dialysis), Feldkirch Academic Teaching Hospital, Feldkirch, Austria; ^2^ Vorarlberg Institute for Vascular Investigation and Treatment (VIVIT), Feldkirch, Austria; ^3^ Agency for Preventive and Social Medicine (aks), Bregenz, Austria

**Keywords:** SARS-CoV-2, kidney transplantation, vaccination, humoral response, SARS-CoV-2 spike protein

Dear Editors,

Solid organ transplant patients are at high risk for severe or fatal COVID-19 (1), even after two vaccinations (2). Recent studies show, that after a double vaccination course, the antibody response rate is as low as 48% (3). However, to our knowledge there is no data on differences in the natural or vaccine-induced SARS-CoV-2 humoral immunity evaluated in one and the same cohort of kidney transplant recipients (KTR). Here, we are reporting on and comparing the humoral response in 164 KTR (mean age 59.1 years (range 21–85 years), 61.6% male). The group included 142 patients who were vaccinated twice (72% Moderna mRNA-1273 vaccine; 27% Pfizer/BioNTech mRNA-BNT162b2 SARS-CoV-2 vaccine, 1% Oxford-AstraZeneca ChAdOx1-COVID-19 vaccine) and 22 patients after symptomatic and PCR-confirmed Covid-19. We assessed the humoral response on average (25th percentile, 75th percentile) 50 days (33.8, 62.0) after the second vaccine dose or 90 days (39.8, 143.0) after infection by quantifying anti-SARS-CoV-2 spike IgG antibodies. Most patients were treated with tacrolimus (74%), mycophenolic acid (71%) and prednisolone (57%). Eight percent were treated with belatacept. Convalescent KTR were significantly younger (*p* = 0.009), had lower eGFR (*p* = 0.021) and were more often treated with prednisolone (*p* = 0.042) as shown in Table 1. Seroconversion was defined as an anti-SARS-CoV-2 IgG antibody concentration above the respective cut-off value according to the manufacturer of the assay. Details about the assays in use and their cut-off values are given in the Supplementary Material.

**TABLE 1:** Characteristics of convalescent and vaccinated kidney transplant patients with and without seroconversion.

	Total (*n* = 164)	Infection (*n* = 22)			Vaccination (*n* = 142)			*p*-value^a^
		Seroconversion (*n* = 20)	No seroconversion (*n* = 2)	Total (*n* = 22)	Seroconversion (*n* = 69)	No seroconversion (*n* = 73)	Total (*n* = 142)	
Age (years), mean (SD)	59.1 (13.9)	51.7 (15.6)	55.0 (1.4)	52.0 (14.9)	59.0 (51.5, 65.0)	62.2 (13.4)	60.2 (13.4)	0.009
Gender (male), *n* (%)	101 (61.6%)	14 (70%)	2 (100%)	16 (72.7%)	44 (63.8%)	41 (56.2%)	85 (59.9%)	0.347
eGFR (ml/min/1.73 m^2^), mean (SD)	52.8 (17.6)	45.0 (14.8)	42.5 (patient 1:49.0; patient 2: 36.0)	44.7 (14.2)	55.2 (16.8)	53.0 (18.8)	54.0 (17.8)	0.021
Transplantation vintage (months), median (25th percentile, 75th percentile)	104.5 (50.3, 174.5)	87.5 (34.0, 151.5)	207.0 (patient 1: 41.0; patient 2: 373.0)	87.5 (38.0, 161.3)	147.0 (93.5, 223.0)	70.0 (37.5, 120.5)	108.5 (53.0, 175.5)	0.361
Time between antigenic contact (infection or vaccination) and antibody assessment (days), median (25th percentile, 75th percentile)		88.0 (37.3, 118.3)	252.5 (patient 1: 230.0, patient 2: 275.0)	90.0 (39.8, 143,0)	51.0 (34.5, 64.0)	43.0 (31.5, 60.0)	50 (33.8, 62.0)	0.001
Immunosuppression, *n* (%)								
Prednisolone	94 (57.3%)	15 (75%)	2 (100%)	17 (77.3%)	30 (43.5%)	47 (64.4%)	77 (54.2%)	0.042
Tacrolimus	121 (73.8%)	17 (85%)	1 (50%)	18 (81.8%)	48 (69.6%)	55 (75.3%)	103 (72.5)	
Mycophenolic acid	116 (70.7%)	15 (75%)	0 (0%)	15 (68.2%)	38 (55.1%)	63 (86.3%)	101 (71.1%)	
Everolimus	1 (0.6%)	0 (0%)	0 (0%)	0	1 (1.4%)	0 (0%)	1 (0.7%)	
Sirolimus	5 (3%)	0 (0%)	1 (50%)	1 (4.5%)	3 (4.3%)	1 (1.4%)	4 (2.8%)	
Belatacept	14 (8.5%)	2 (10%)	0 (0%)	2 (9.1%)	1 (1.4%)	11 (15.1%)	12 (8.5%)	
Cyclosporin A	23 (14.0%)	2 (10%)	0 (0%)	2 (9.1%)	16 (23.2%)	5 (6.8%)	21 (14.8%)	
Azathioprine	16 (9.8%)	2 (10%)	1 (50%)	3 (13.6%)	11 (15.9%)	2 (2.7%)	13 (9.2%)	

a *p*-value for group comparison infection *versus* vaccination.

The seroconversion rate in convalescent patients was 90.9 and 48.6% in vaccinated patients (*p* < 0.001). In the patients treated with belatacept, only one out of 12 (8.3%) vaccinated individuals had a seroconversion, whereas both naturally infected patients showed a response. In a multivariable logistic regression analysis infection compared to vaccination [odds ratio (OR) 18.98; 95% CI: 3.41, 105.58] and transplantation vintage (OR 1.01; 95% CI: 1.01, 1.02) were associated with a significantly higher likelihood of seroconversion. On the other hand, older age (OR 0.95; 95% CI: 0.93, 0.99) and belatacept treatment (OR 0.13; 95% CI: 0.02, 0.68) significantly decreased the likelihood of seroconversion. All model coefficients and odds can be found in the Supplementary Table S1.

Like our results in convalescent KTR, Magicova et al. recently found a preserved humoral response after SARS-CoV-2 infection comparable to immunocompetent persons in a large Czech cohort of 1,037 kidney transplant recipients with a seroprevalence of 6.8% during the second infection wave in fall 2020 (4). In line with our findings, recent data in dialysis patients also show a superior humoral immune response in convalescent compared to vaccinated patients (5). Natural infection seems to be a stronger and quantitatively higher antigenic challenge than vaccination. In contrast to intramuscular vaccination, natural infection stimulates the resident immune system of mucous membranes, especially designed to fight respiratory viral diseases. These considerations and first experience showing a 49–70% humoral response rate after a third vaccine dose in kidney transplant patients without seroconversion after two doses (6–8) support an early third vaccination to improve the seroconversion rate in this vulnerable population.

## Data Availability

The original contributions presented in the study are included in the article/Supplementary Material, further inquiries can be directed to the corresponding author.
