# Supplementary material for: Humoral Response in SARS-CoV-2 Convalescent Compared to Vaccinated Kidney Transplant Patients
Source: Transpl Int. 2022 Jan 18;35:10060. doi: 10.3389/ti.2021.10060 (PMC8842381; doi:10.3389/ti.2021.10060)
Supplement: Supplementary file 1 [file DataSheet1.docx]

**Supplementary Material**

**Table of content**

**Study Design and Population**

**Supplementary Methods**

**Supplementary Table S1.** Binomial logistic regression analysis to predict the likelihood of seroconversion

**Supplementary References**

**Study Design, Population and Methods**

For the present retrospective study 164 kidney transplant patients were recruited in one center (Feldkirch Academic Teaching Hospital) in Vorarlberg, the westernmost state of Austria, caring for 262 kidney transplant patients. Twenty-two patients had a PCR-confirmed infection with SARS-CoV-2. The majority of infections were mild. Eleven patients were hospitalised, with two in intensive care and on ventilation and three on oxygen supplementation. One hundred and forty-two patients had no infection but had been fully vaccinated (72% Moderna mRNA-1273 vaccine; 27% Pfizer/BioNTech mRNA-BNT162b2 SARS-CoV-2 vaccine, 1% Oxford–AstraZeneca ChAdOx1-COVID-19 vaccine). No serious adverse events after vaccination were reported. Median time since transplantation (transplantation vintage) until antigenic contact (infection or first vaccination) was 101 months (range 1-433 months). Serum anti-SARS-CoV-2 IgG levels were analysed in 78.7% of patients with the LIAISON SARS-CoV-2 TrimericS IgG Assay (Diasorin S.p.A., Saluggia, Italy; seroconversion:  ≥33.8 BAU/mL) detecting IgG antibodies to the trimeric complex spike glycoprotein including the receptor-binding domain (RBD) and N-terminal domain (NTD) of the S1 subunit, in 6.7 % with the Elecsys Anti-SARS-CoV-2 Spike protein Assay (Roche, Rotkreuz, Switzerland; seroconversion: ≥0.82 BAU/ml) detecting IgG antibodies to the RBD, in 5.5% with the SARS-CoV-2 IgG II Assay (Abbott, Abbott Park, Illinois, U.S.A; seroconversion: index value ≥1.40) detecting IgG antibodies to the RBD, in 4.9% (all of them patients after PCR-confirmed infection) with the Platelia SARS-CoV-2 Total Ab Assay (Bio-Rad Laboratories Ltd., Hertfordshire, England; seroconversion: ratio ≥0.8) detecting antibodies against the nucleocapsid protein, in 2.4% with the ELISA Kit (TECOmedical AG,Sissach, Switzerland; seroconversion:  ≥20.0 IU/mL) detecting IgG antibodies directed against the RBD, and in 1.8% with the cPass™ SARS-CoV-2 Neutralization Antibody Detection Kit (GenScript, Leiden, the Netherlands; seroconversion:  ≥30%) detecting neutralizing IgG antibodies to the RBD. Therefore, absolute antibody titers were not reported and compared statistically due to several different test kits. The study was conducted in compliance with the Helsinki Declaration of 1975, as revised in 2013, and Good Clinical Practice. Patients provided written informed consent. Ethical committee approval was not necessary due to the retrospective design.

Data sharing statement: All data is included in the manuscript and will be shared by the corresponding author on reasonable request.

**Statistical analyses**

Categorical data are presented as absolute and relative number of patients. For continuous data mean and standard deviation (SD) or median with interquartile range (25^th^ percentile, 75^th^ percentile) is used, depending on its distribution. Categorical parameters were compared using Chi-squared or Fisher´s exact tests, continuous parameters were analysed with Student's T test or with Mann-Whitney U test depending on data distribution. A multivariable binomial logistic regression was performed to determine the effect of gender, age, mode of antigen contact (infection vs vaccination), eGFR, transplantation vintage, prednisolone and belatacept treatment and predict the likelihood of achieving seroconversion. Linearity was tested assessed using the Box-Tidwell procedure^S1^, Bonferroni-correction was applied to all terms in the model^S2^. All variables were found to follow a linear relationship. Correlations between predictor variables were low (r <0.35), indicating that multicollinearity was not a confounding factor in the analysis. The binomial logistic regression model was statistically significant, χ²(7) = 57.693, p <0.001. Goodness-of-fit was assessed using the Hosmer-Lemeshow-Test, indicating a good model fit, χ²(8) = 5.709, p =0.680. A two-sided P value <0.05 was deemed to indicate statistical significance. All statistical analyses were performed with IBM SPSS Statistics 27 (IBM, Armonk (NY), USA).

**Supplementary Table S1. Binomial logistic regression analysis to predict the likelihood of seroconversion**

|  | | **B** | **SE** | **Wald** | **P** | **Odds Ratio** | **95% CI for Odds Ratio** | |
| --- | --- | --- | --- | --- | --- | --- | --- | --- |
|  |  |  |  |  |  |  | **Lower Bound** | **Upper Bound** |
|  | Gender (male *vs* female) | 0.499 | 0.393 | 1.614 | 0.204 | 1.647 | 0.763 | 3.556 |
|  | Age (years) | -0.047 | 0.016 | 8.570 | 0.003 | 0.954 | 0.925 | 0.985 |
|  | eGFR (ml/min/1.73 m²) | 0.010 | 0.012 | 0.753 | 0.385 | 1.010 | 0.987 | 1.034 |
|  | Transplantation vintage (months) | 0.012 | 0.003 | 17.184 | <0.001 | 1.012 | 1.006 | 1.017 |
|  | Antigen contact (infection *vs* vaccination) | 2.943 | 0.876 | 11.302 | 0.001 | 18.982 | 3.413 | 105.582 |
|  | Prednisolone (yes *vs* no) | -0.237 | 0.439 | 0.290 | 0.590 | 0.789 | 0.334 | 1.867 |
|  | Belatacept (yes *vs* no) | -2.081 | 0.865 | 5.788 | 0.016 | 0.125 | 0.023 | 0.680 |
|  | Constant | 0.746 | 1.389 | 0.288 | 0.591 | 2.108 |  |  |

**Supplementary References**

^S1^Box GEP, Tidwell PW. Transformation of the Independent Variables. *Technometrics* 1962; **4(4):** 531–550.

^S2^Tabachnick BG, Fidell LS. *Using Multivariate Statistics* 2018 (7th ed.): Pearson Education.
